# Calibration of contact parameters of sandy soil for planting tiger nut based on non-linear tools

**DOI:** 10.1038/s41598-024-64635-y

**Published:** 2024-06-17

**Authors:** Jiangtao Qi, Sirui Chen, Luoyi Yang, Shiguan An, Hewei Meng, Za Kan

**Affiliations:** 1 College of Mechanical and Electrical Engineering, Shihezi University, Xinjiang, 832000 China; 2 Engineering Research Center for Production Mechanization of Oasis Special Economic Crop, Ministry of Education, Xinjiang, 832000 China; 3 Key Laboratory of Northwest Agricultural Equipment, Ministry of Agriculture and Rural Affairs, Xinjiang, 832000 China

**Keywords:** Discrete element, Parameter calibration, RSM, Sandy soil, WNN algorithm, Electrical and electronic engineering, Mechanical engineering, Actuators

## Abstract

A methodology combining physical experiments with simulation was employed to acquire contact parameters of sandy soil precisely for planting tiger nuts in the desert area of Xinjiang. The stacking angle under different parameter combinations was applied as a response value. Through the Plackett–Burman test, several factors that have a significant influence were determined. The steepest ascent test was conducted to establish the finest scope of values for these parameters. The stacking angle was considered the response variable, and non-linear tools were used to optimize these parameters for simulation. The findings showed that applying response surface methodology (RSM) resulted in a relative error of 1.24%. In the case of BP-GA, the relative error compared to the physical test value was 0.34%, while for BP, it was 2.18%. After optimization using Wavelet Neural Network (WNN), the relative error was reduced to only 0.15%. Results suggest that WNN outperforms the RSM model, and the sandy soil model and parameters generated using WNN can be effectively utilized for discrete element simulation research.

## Introduction

In China, Xinjiang has the most extensive range and most immense expanse of deserts. The total desert area in Xinjiang spans approximately 420,000 km^2^, accounting for 26.12% of the province's total area^1^. In recent years, the domestic desert industry has experienced significant growth, and Xinjiang has made notable achievements in its development^2^. However, the industrialization of certain economic crops in desert regions has yet to catch up with the overall progress of the desert industry. For instance, sowing and harvesting crops like tiger nuts need more suitable mechanical equipment, leading to low production efficiency and high labor costs. The above things, in turn, hinder the development of Xinjiang's desert industry. Consequently, it becomes crucial to study and develop mechanical equipment that can mechanize the production of economic crops in Xinjiang's desert areas. The first step in researching and developing such equipment involves gaining a comprehensive understanding of the characteristics of sandy soil for planting economic crops in Xinjiang.

With the progress in high-tech, Discrete Element Methodology (DEM) has gained popularity in investigating bulk materials^3,4^. The discrete element method is a numerical simulation method specifically designed to solve problems in discontinuous media. The basic principle of this method is to treat the cleavage material as a discrete material and the cleavage surface between materials, allowing for material translation, rotation, and deformation. In contrast, the cleavage surface can be compressed, separated, or slid. It can have large displacement, rotation, sliding, and even block separation inside, which can simulate the nonlinear prominent deformation characteristics in cleavage materials more realistically. DEM enables a comprehensive and systematic examination of the motion characteristics of bulk materials. It not only facilitates the optimization of structural and operational parameters of mechanical devices but also enhances development efficiency, improves mechanical performance, and reduces costs. Parameter calibration is a valuable approach to accurately obtaining material characteristic parameters in material characteristic research. Currently, scholars in related research fields have conducted studies on parameter calibration of agricultural bulk materials adopting DEM. For example, Zhang et al.^5^ introduced a technique for calibrating interaction parameters between sand and soil using a combined approach, and examined the effects of varying masses and calibration methods on the repose angle. The findings revealed that the particle morphology has a noteworthy influence on the static friction coefficient. Wu^6^ employed orthogonal experimental methods to calibrate the interaction parameters among sand particles. The simulated deposition angle was determined to be 26.586°, exhibiting a relative error of 1.22%, thus confirming the accuracy of the simulation outcomes. Wang^7^ investigated the calibration of contact parameters among sand particles, with findings indicating that the variance between virtual simulation and actual testing was less than 5.1%. Wu^8^, Lin^9^, and Wang^10^ analyzed and calibrated the contact parameters of manure. All three studies confirmed the viability of the methodologies, thereby yielding commendable experimental outcomes. Coetzee^11^ and Pasha^12^ demonstrated that particle models of various shapes necessitated parameters during the simulation, which should be calibrated via experimentation. Moreover, Liu, Wang, and Liu^13–15^ calibrated contact parameters of crops such as wheat, corn, and mini-potatoes, respectively, providing valuable references for parameter optimization and performance analysis of crop harvesting mechanization. The above studies have all adopted the discrete element calibration method, which allows for relative motion between individual cells, does not necessarily meet the conditions of continuous displacement and deformation coordination, has fast calculation speed, and requires small storage space to solve problems with large displacement and nonlinearity, and has achieved promising experimental results.

Furthermore, machine learning techniques have found extensive applications in engineering. For instance, references^16–18^ demonstrate modeling using the cosine amplitude method, and relevant algorithms were then employed to analyze and predict various engineering issues. Simultaneously, numerous machine learning studies to solve engineering problems have proposed several valuable and innovative designs^19–22^.

The studies mentioned above have yielded promising results; however, there is still scope for further improvement. Based on the study mentioned above, this research focuses on sandy soil found in the desert areas of Xinjiang, where tiger nuts are cultivated. A combined approach was utilized to calibrate the contact parameters of sandy soil's discrete elements^23^. Afterward, the obtained crucial parameters were optimized and fitted by applying non-linear tools, followed by the acquisition and comparison of predicted values. Consequently, a model was developed, serving as a conceptual background for the design of devices in the mechanized production of economic crops like tiger nuts.

## Materials and methods

### Materials

The sandy soil was acquired from a tiger nut plantation in the 54th Regiment of the Third Division in Xinjiang. The 54th Regiment is in Shache County, on the southwestern frontier of Xinjiang, at the northern foothills of the Kunlun Mountains, and south of the Pamir Plateau. It lies within the mid-upper reaches of the Yarkand River alluvial fan plain, positioned between the Taklamakan Desert and the Bugur Desert, with the terrain sloping from west to east. The geographical coordinates are approximately between 76°1′57″ to 77°46′30″ E longitude and 37°27′30″ to 39°15ʹ N latitude. A bulk density meter was employed to determine its density. The standard for the determination of the particle size distribution is GB/T 50123-2019. The particle size distribution was determined by screening soil by applying a standard particle size sieve. Additionally, moisture content was measured, as presented in Table 1. The intrinsic parameters for sandy soil and steel plate were determined based on previous studies^6,24–26^ and the GEMM database of EDEM, as listed in Table 2.

**Table 1:** Fundamental elements of sandy soil.

Size distribution/(%)			Density/(g/cm^−3^)	Moisture/(%)
> 0.5 mm	(0.25–0.5] mm	≤ 0.25 mm		
0.36	7.45	92.29	2.92	6.4

**Table 2:** Intrinsic parameters of sandy soil and steel plate.

Material	Poisson's ratio	Shear modulus	Density(g/cm^−3^)
Sandy soil	0.3	1.15 × 10^7^	2.92
Steel plate	0.3	7.94 × 10^10^	7.85

As an intrinsic characteristic of bulk materials, stacking angle can provide insights into their flow and friction properties^27–29^. A combinatorial approach^11^ was employed to calibrate parameters. Injection methodology was utilized to measure the actual stacking angle. Plackett–Burman design, conducted using Design-Expert 8.06, was applied for a multi-factor significance screening test and analysis to identify parameters that have a crucial influence on stacking angle. Box-Behnken design RSM was applied to form and ameliorate a regression model. Compared to the central composite design, the Box-Behnken design requires fewer trials and typically features a more uniform layout of test points. Additionally, it excludes pivot points on the boundaries of the test range, thereby reducing the extreme conditions of the experiment. Therefore, this study opted for the Box-Behnken design. The actual stacking angle was adopted as the objective value. A regression model was also used to determine the combination of crucial parameters. Subsequently, a simulation was performed by applying optimal parameters. The simulated value was contrasted with the actual value to validate the validity of the calibrated parameters.

### Physical model

**Figure 1:**
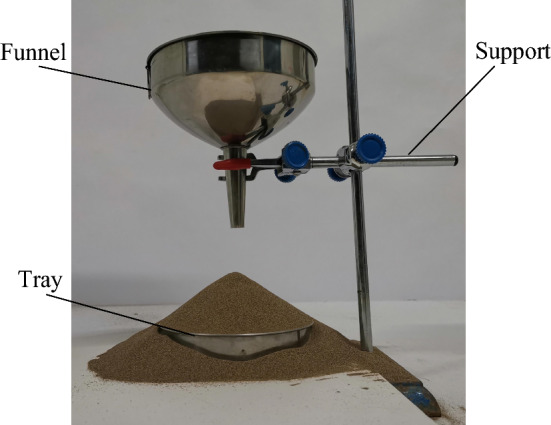
Physical test of stacking angle.

**Figure 2:**
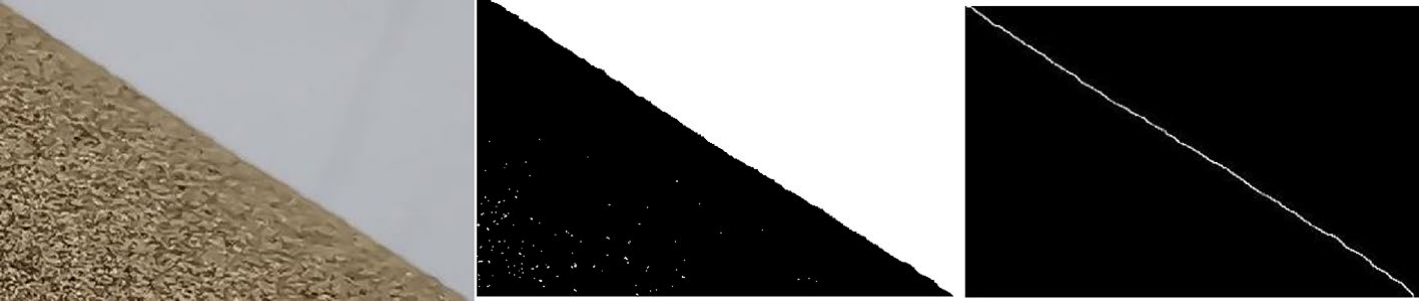
Test processing.

Based on the related investigations in the paper^30–32^, injection methodology was adopted following GB 11986-89/ISO 4324-1977. The measuring device is depicted in Fig. 1. The lower mouth of the funnel had an inner diameter of 10 mm, while the funnel itself had a taper angle of 60°. The tray, with a diameter of 100 mm, had a height of 25 mm. The distance between the lower mouth and the upper surface of the tray was measured to be 75 mm. During the procedure, sandy soil particles in the funnel fell on the tray through the funnel mouth and formed a steady pile of particles. First, the stacking angle image was captured from the side. This image was then imported into MATLAB software for processing to obtain the angle. The experiment was conducted five times. The mean calculated value of the stacking angle was computed as 32.15°. The results of the physical experimental equipment are shown in Figs. 1 and 2.

### Simulation model configuration

#### Contact model betwixt particles

The hertz-Mindlin (no slip) contact model is known for its accuracy in force calculation. This model proves highly efficient given the minimal cohesive action betwixt particles in sandy soil^33^.

Assuming that two particles with radii *R*_*1*_ and *R*_*2*_ are brought into elastic contact, the mathematical calculation for normal overlap *α* is

1 $$\alpha = R_{1} + R_{2} - \left| {{\vec{\text{r}}}_{{1}} - {\vec{\text{r}}}_{{2}} } \right|$$

${\vec{\text{r}}}_{{1}}$ and ${\vec{\text{r}}}_{{2}}$ are sphere center position vector.

Contact radius of contact surface is $a$.

2 $$a = \sqrt {\alpha R^{ * } }$$

$R^{ * }$ is equivalent particle radius, which is computed as follow,

3 $$\frac{{1}}{{R^{ * } }} = \frac{{1}}{{R_{1} }} + \frac{1}{{R_{2} }}$$

Normal force betwixt particles $F_{n}$ is

4 $$F_{n} = \frac{4}{3}E^{ * } (R^{ * } )^{{{\raise0.7ex\hbox{$1$} \!\mathord{\left/ {\vphantom {1 2}}\right.\kern-0pt} \!\lower0.7ex\hbox{$2$}}}} \alpha^{{{\raise0.7ex\hbox{$3$} \!\mathord{\left/ {\vphantom {3 2}}\right.\kern-0pt} \!\lower0.7ex\hbox{$2$}}}}$$

$E^{ * }$ is equivalent elastic modulus, which is computed as follow,

5 $$\frac{{1}}{{E^{ * } }} = \frac{{1 - \nu_{1}^{2} }}{{E_{1} }} + \frac{{1 - \nu_{2}^{2} }}{{E_{2} }}$$

where, $E_{1}$ represents the elastic modulus of Particle 1, $\nu_{1}$ denotes the Poisson's ratio of Particle 1, $E_{2}$ represents the elastic modulus of Particle 2, and $\nu_{{2}}$ denotes the Poisson's ratio of Particle 2.

Normal damping force can be acquired by later calculation.

6 $$F_{{\text{n}}}^{{\text{d}}} = { - 2}\sqrt {\frac{{5}}{{6}}} \beta \sqrt {S_{{\text{n}}} {\text{m}}^{ * } } \upsilon_{{\text{n}}}^{{{\text{rel}}}}$$

m* means equivalent mass, which is acquired as follow,

7 $$m^{*} = \frac{{m_{1} m_{2} }}{{m_{1} + m_{2} }}$$

8 $$\beta = \frac{\ln e}{{\sqrt {\ln^{2} e + \pi^{2} } }}$$

9 $$S_{n} = 2E^{*} \sqrt {R^{*} S_{n} }$$

where, $\beta$ is damping ratio, $e$ is restitution factor, $\upsilon_{{\text{n}}}^{{{\text{rel}}}}$ is normal component of relative velocity, and $S_{{\text{n}}}$ means normal stiffness.

10 $$F_{s}^{\tau } = - S_{\tau } \delta_{\tau }$$

11 $$S_{\tau } = {8}G^{ * } \sqrt {R^{ * } \delta_{n} }$$

$S_{\tau }$ means tangential stiffness. $\delta_{\tau }$ is tangential overlap. Besides, $G^{ * }$ means equivalent shear modulus.

12 $$F_{d}^{\tau } = - 2\sqrt{\frac{5}{6}} \beta \sqrt {S_{\tau } m^{ * } } \upsilon_{\tau }^{rel}$$

$\upsilon_{\tau }^{rel}$ means tangential component of relative velocity.

Rolling friction be described as,

13 $$T_{{\text{i}}} = { - }\mu_{{\text{r}}} F_{{\text{n}}} R_{{\text{i}}} \omega_{{\text{i}}}$$

where, $\mu_{{\text{r}}}$ means rolling friction coefficient, $R_{{\text{i}}}$ represents distance from contact point to center of mass. In addition, $\omega_{{\text{i}}}$ is unit angular vector of object at contact point.

#### Establishment of geometric model

To establish a suitable particle model, the mass distribution of sandy soil particles ranging from 0 to 0.25 mm was scrutinized, and it was found that they accounted for 92.29% of the total mass among the selected test samples, making them representative of the overall sand sample. Therefore, these particles were selected for modeling purposes. It should be noted that the size and shape of the sand particle impact the results and the computational efficiency of the computer. Considering laboratory conditions and physical properties of sand particles, spherical particles with a size of 1 mm were selected.

**Figure 3:**
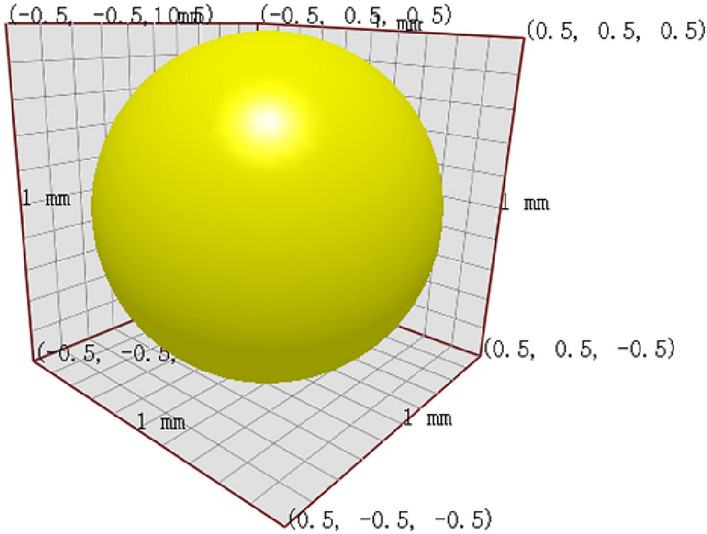
Particle model.

**Figure 4:**
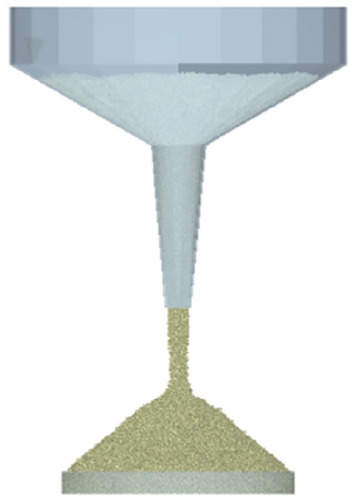
Funnel model.

Using a physical test model as a basis, a model was created in SolidWorks software, which was integrated into EDEM as a simulation model for the funnel device, utilizing the Hertz-Mindlin (no slip) contact model. Figures 3 and 4 display particle model and funnel model, respectively.

It is determined that the initial radius of particles generated in the simulation procedure should be 1 mm, and they were randomly generated. To prevent the generation of tiny particles, the radius of the generated particles was limited to between 0.5 and 1.25 times the initial radius.

Dynamic generation of particles in the funnel was performed using a virtual particle factory above the funnel. The overall weight produced was 0.45 kg, with a rate of 0.2 kg/s. The time interval for data storage was established as 0.01 s, while the fixed time step was 20% of the Rayleigh time step. Furthermore, the mesh size was three times the smallest spherical element size^6,34,35^. Values of the other parameters are shown in Table 3.

**Table 3:** Simulation parameters.

Parameter	Numerical value
Sandy soil-sandy soil restitution coefficient	0.1–0.7
Sandy soil-sandy soil static friction coefficient	0.2–0.8
Sandy soil-sandy soil rolling friction coefficient	0.1–0.3
Sandy soil-steel restitution coefficient	0.1–0.6
Sandy soil-steel static friction coefficient	0.2–0.8
Sandy soil-steel rolling friction coefficient	0.04–0.4

### Test methodology

#### RSM test methodology

##### Plackett Burman design (PBD) screening experiment

A PBD screening experiment was adopted to ascertain the importance of every individual factor. The test examines the correlation between objective response and every individual factor, contrasting the difference between the two levels of every individual factor. The stacking angle of sandy soil was selected as the response value for screening contact parameters. Two levels were chosen for every individual of the six factors. Simulation parameters were determined based on characteristics of sandy soil used for planting economic crops in desert areas of Xinjiang. Table 4 shows levels of factors, along with corresponding symbols.

**Table 4:** PBD.

Symbol	Factor	Level	
		− 1	1
A	Sandy soil-sandy soil restitution coefficient	0.3	0.6
B	Sandy soil-sandy soil static friction coefficient	0.4	0.8
C	Sandy soil-sandy soil rolling friction coefficient	0.1	0.3
D	Sandy soil-steel plate restitution coefficient	0.3	0.6
E	Sandy soil-steel plate static friction coefficient	0.2	0.4
F	Sandy soil-steel plate rolling friction coefficient	0.2	0.4
G ~ L	Dummy parameter	–	–

##### Steepest ascent test

The steepest ascent test is a methodology that can quickly identify the area with optimal response value while minimizing the number of tests required. It was conducted by increasing or decreasing values of significant parameters according to predetermined step length^36^. For residual parameters, the median values were selected. Specifically, A was 0.4, C was 0.2, and F was 0.22. The test scheme can be found in Table 7, and the relative error (*N*, %) is shown in the formula (14).

14 $$N = \frac{{\left| {\sigma { - }\theta } \right|}}{\theta } \times {\text{100\% }}$$

where, $\sigma$ is stacking angle of simulation test (°). $\theta$ is stacking angle of physical test (°).

##### Box Behnken design response surface experiment

Based on the principle of response surface design^37^, the Box-Behnken test was conducted with three levels of significant parameters (low, medium, and high). Additionally, five central points were included to estimate the error. Factor values are provided in Table 5. Factors corresponding to each symbol are consistent with Table 4.

**Table 5:** Parameter value of Box-Behnken experiment.

Factors	Range		Value
	− 1	1	
A	0.1	0.7	0.4
B	0.5	0.7	0.2–0.8
C	0.1	0.3	0.2
D	0.4	0.6	0.4–0.6
E	0.3	0.5	0.3–0.5
F	0.04	0.4	0.22

#### Machine learning algorithm

The Back Propagation (BP) algorithm is a feedforward learning algorithm that utilizes a multi-layer perceptron, also known as BP neural network, to perform backpropagation. This algorithm is iterative and involves forward propagation, backpropagation, and parameter updates. On the other hand, Genetic Algorithms (GA) offer advantages such as efficiency, parallelism, and global search. Normalization processing must be performed before using the BP-GA integrated optimization algorithm for data training. This is followed by selecting one feature value as the output and the remaining feature values as the input. Upon completion of training, the BP-GA integrated optimization algorithm generates prediction results.

**Figure 5:**
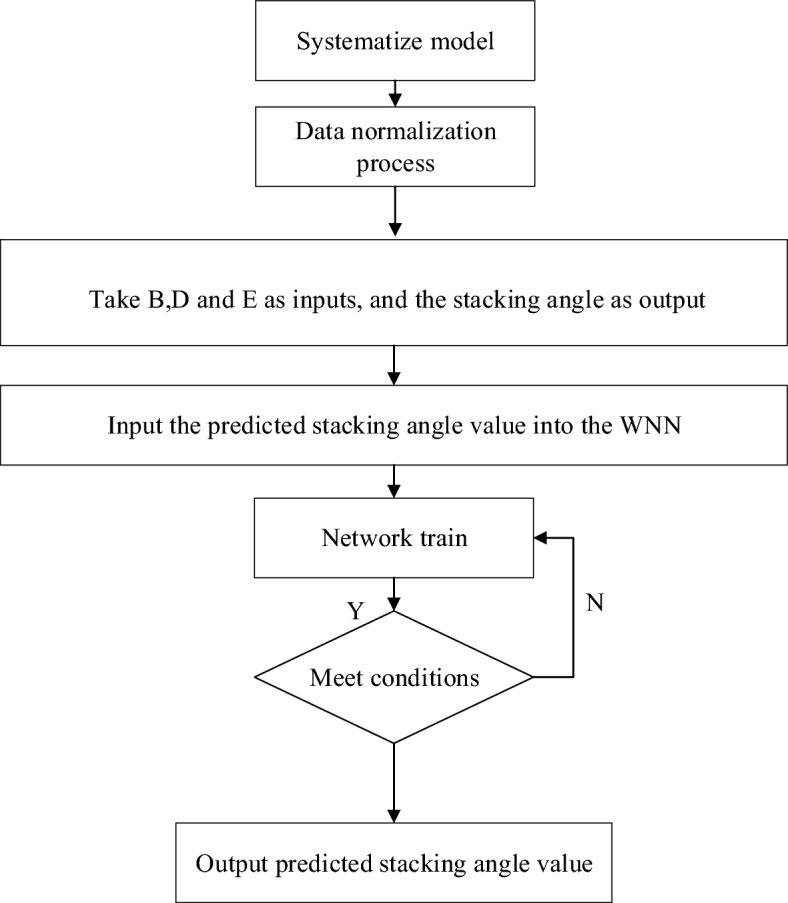
WNN flowchart.

Wavelet Neural Network (WNN) is constructed based on the wavelet transform theory, and its principle is similar to that of the Backpropagation Neural Network (BPNN). The primary characteristic of WNN is that its hidden layer neuron activation function is a wavelet basis function, which effectively utilizes the localization property of wavelet transform and the large-scale data parallel processing and self-learning capability of neural networks. Consequently, it exhibits a vital ability for approximation and achieves fast convergence speed. The specific flowchart is shown in Fig. 5.

Train and optimize stacking angle using the WNN optimization approach. Before commencing the experiment, three sets of data were randomly chosen from every amalgamation of factor levels, leading to a total of 51 data sets being extracted. WNN was employed to learn and train these 51 data sets as training sets. Subsequently, the algorithm's performance in predicting stacking angle was assessed using 17 other test sets, which can be seen in the table.

**Figure 6:**
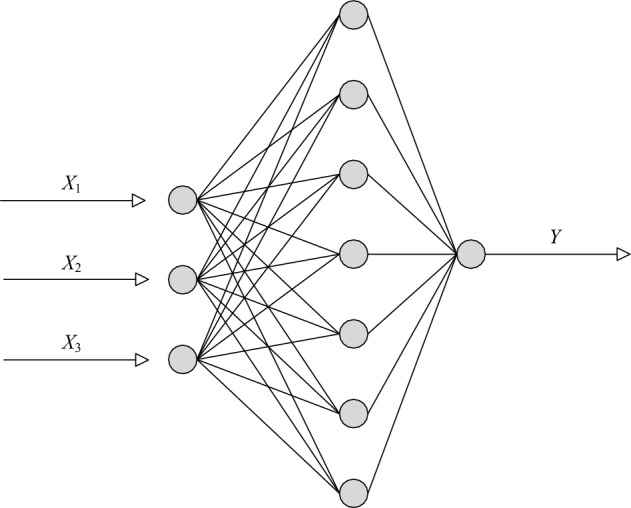
Topological diagram of WNN structure.

Applying WNN to predict the stacking angle response value involves predicting the dependent variable by considering the explanatory variables. Previous experiments have identified B, D, and E as the three most significant influencing factors, and this experiment employs them as explanatory variables. *X*_*1*_, *X*_*2*_, and *X*_*3*_ were used as input, and *Y* was used as the output (as shown in the topology diagram in Fig. 6). B, D, and E were represented by the numbers 1, 2, and 3, respectively. To enhance training outcomes, it is essential to normalize the training sample set in the preprocessing stage, considering the significant disparities in the magnitude of each input variable. The objective of this normalization process is to attain superior training results.

## Simulation experiment and result analysis

### PBD screening test

Table 6 displays the design and outcomes of PBD. ANOVA analysis was conducted on outcomes applying Design Expert 8.06^38^, and the significance of parameters is displayed in Table 5, which reveals that factors A, B, and D exhibit a positive repercussion, indicating that stacking angle enhances as these factors enhance. Conversely, factors C, E, and F have a negative effect. The significance sequence of every factor was ascertained based on the P value of factors shown in Table 7. Three factors, E, D, and B, which have a higher significance, were selected in the steepest ascent experiment and the Box-Behnken design experiment.

**Table 6:** Scheme and results of PBD.

No	Test factor											Stacking angle/(°)
	A	B	C	D	E	F	G	H	J	K	L	
1	− 1	− 1	1	− 1	1	1	− 1	1	1	1	− 1	22.29
2	− 1	1	1	1	− 1	− 1	− 1	1	− 1	1	1	32.21
3	1	1	− 1	− 1	− 1	1	− 1	1	1	− 1	1	30.11
4	1	1	− 1	1	1	1	− 1	− 1	− 1	1	− 1	29.68
5	1	− 1	1	1	− 1	1	1	1	− 1	− 1	− 1	29.25
6	− 1	1	− 1	1	1	− 1	1	1	1	− 1	− 1	27.02
7	1	1	1	− 1	− 1	− 1	1	− 1	1	1	− 1	27.79
8	− 1	1	1	− 1	1	1	1	− 1	− 1	− 1	1	23.75
9	1	− 1	− 1	− 1	1	− 1	1	1	− 1	1	1	24.23
10	1	− 1	1	1	1	− 1	− 1	− 1	1	− 1	1	27.02
11	− 1	− 1	− 1	− 1	− 1	− 1	− 1	− 1	− 1	− 1	− 1	25.91
12	− 1	− 1	− 1	1	− 1	1	1	− 1	1	1	1	25.64

*G, H, J, K and L indicates blank column.*

**Table 7:** Plackett Burman experiment results.

Source of variance	Effects	Sum of squares	*P* value	Significance
A	1.88	10.57	0.0937	4
B	2.70	21.92	0.0309	3
C	− 0.047	6.53 × 10^–3^	0.9610	6
D	2.79	23.35	0.0277	2
E	− 2.82	23.86	0.0267	1
F	− 0.58	1.00	0.5534	5

### Steepest climb test result analysis

Findings from the steepest ascent test are displayed in Table 8. Findings revealed that the stacking angle gradually enhanced as factors B, D, and E lessened. Furthermore, the relative error between the actual stacking and initial angles was lessened initially and then enhanced. The most minor relative error was observed in the No. 5 test. Consequently, parameter values from the No. 5 test were chosen as central points for subsequent tests. In contrast, parameter values from the No. 4 and No. 6 tests were considered low and high levels for subsequent response surface design.

**Table 8:** Scheme and results of the steepest ascent experiment.

No	Parameters			Stacking angle/(°)	Relative errors/%
	B	D	E		
1	0.2	0.1	0.8	23.56	26.7
2	0.3	0.2	0.7	25.78	19.8
3	0.4	0.3	0.6	26.57	17.4
4	0.5	0.4	0.5	28.5	11.4
5	0.6	0.5	0.4	31.76	1.2
6	0.7	0.6	0.3	35.71	11.1

### RSM analysis test

The experimental plan comprises 17 test points, encompassing 12 analysis elements and 5 zero estimation errors.

Box-Behnken experiment results, shown in Table 9, indicate that three significant parameters investigated, i.e., B, D, and E. Design Expert 8.06, were applied to find a quadratic regression equation. The equation is as follows,

15 $$\theta = 31.53 + 1.63B + 1.41D - 0.93E + 0.77BD - 0.95BE - 0.48DE - 0.47B^{2} - 0.7D^{2} - 0.39E^{2}$$

**Table 9:** Design and results of Box-Behnken.

No	B	D	E	Stacking angle/(°)	BP	BP-GA	WNN
1	− 1	− 1	0	28.51	28.54	28.52	28.51
2	1	− 1	0	30.67	30.64	30.65	30.66
3	− 1	1	0	29.76	29.52	29.60	29.75
4	1	1	0	35.02	35.04	35.03	34.99
5	− 1	0	− 1	29.59	29.53	29.54	29.55
6	1	0	− 1	34.30	34.31	34.30	34.30
7	− 1	0	1	28.94	29.76	29.78	29.92
8	1	0	1	29.85	29.86	29.85	29.80
9	0	− 1	− 1	29.76	29.52	29.60	29.75
10	0	1	− 1	33.55	33.54	33.55	33.53
11	0	− 1	1	29.56	28.92	28.93	29.54
12	0	1	1	31.43	31.45	31.42	31.43
13	0	0	0	31.96	31.45	31.53	31.85
14	0	0	0	31.43	31.46	31.45	31.43
15	0	0	0	30.97	31.53	31.53	30.89
16	0	0	0	31.82	31.47	31.53	31.51
17	0	0	0	31.49	31.54	31.51	31.94

ANOVA results are displayed in Table 10. For fitting the mathematical model, *P* = 0.0003, revealing an excellent fitting degree. *P* value of B was less than 0.01, *P* values of D, E, interaction item B × D, and interaction item B × E were all less than 0.05, confirming their significance on stacking angle and validating regression. For Lack of Fit, *P* = 0.1529 > 0.05, revealing a tiny proportion of atypical errors in fitting betwixt obtained regression equation and actual value, with a splendid fitting degree without lack-of-fit phenomenon. The coefficient of variation *CV* = 1.7% indicated high validity. The coefficient of determination *R*^*2*^ = 0.964 and adjusted coefficient of determination *R*^*2adj*^ = 0.918, all-surpassing 0.9, demonstrating that the proposed model precisely represents an actual situation. Additionally, *Adep Precision* = 14.948, demonstrating the proposed model's high precision.

**Table 10:** ANOVA results of Box-Behnken experiment.

Source of variance	Mean squares	Degrees of freedom	Sum of square	Ratio of *F*	*P* value
Model	52.65	9	5.85	20.83	0.0003
B	21.26	1	21.26	75.69	< 0.0001
D	15.85	1	15.85	56.44	0.0001
E	6.88	1	6.88	24.51	0.0017
B × D	2.4	1	2.4	8.56	0.0222
B × E	3.61	1	3.61	12.86	0.0089
D × E	0.92	1	0.92	3.28	0.1129
B^2^	0.95	1	0.95	3.38	0.1088
D^2^	0.02	1	0.02	0.072	0.7956
E^2^	0.64	1	0.64	2.27	0.1752
Residual	1.97	7	0.28		
Lack of Fit	1.37	3	0.46	3.08	0.1529
Pure Error	0.59	4	0.15		
Sum	54.62	16			
*R*^*2*^ = 0.964 *R*^*2adj*^ = 0.918 *CV* = 1.7% *Adep precision* = 14.948					

According to the results in Table 11, factors that have no significant impact (D × E, B^2^, D^2^, and E^2^) were eliminated. ANOVA results of the modified mathematical model are displayed in Table 11. For fitting mathematical model, *P* < 0.001; for Lack of Fit, *P* = 0.1043; coefficient of variation *CV* = 2.08%; determination coefficient *R*^*2*^ = 0.915; adjusted determination coefficient *R*^*2adj*^ = 0.877; test precision *Adep Precision* = 15.969. It reveals that the proposed model had a splendid fit, validity, and accuracy and had specific improvements that were in contrast with those before optimization. After optimization, the regression formula is,

16 $$\theta = 31.09 + 1.63B + 1.41D - 0.93E + 0.77BD - 0.95BE$$

**Table 11:** ANOVA of modified model of Box-Behnken experiment.

Source of variance	Sum of square	Degrees of freedom	Mean squares	Ratio of *F*	*P* value
Model	50	5	10	23.81	< 0.0001
B	21.26	1	21.26	50.62	< 0.0001
D	15.85	1	15.85	37.74	< 0.0001
E	6.88	1	6.88	16.39	0.0019
B × D	2.4	1	2.4	5.72	0.0357
B × E	3.61	1	3.61	8.6	0.0136
Residual	4.62	11	0.42		
Lack of Fit	4.02	7	0.57	3.87	0.1043
Pure Error	0.59	4	0.15		
Sum	54.62	16			
*R*^*2*^ = 0.915 *R*^*2adj*^ = 0.877 *CV* = 2.08% *Adep precision* = 15.969					

### Analysis of interaction effects in regression models

#### Analysis of interaction effects

**Figure 7:**
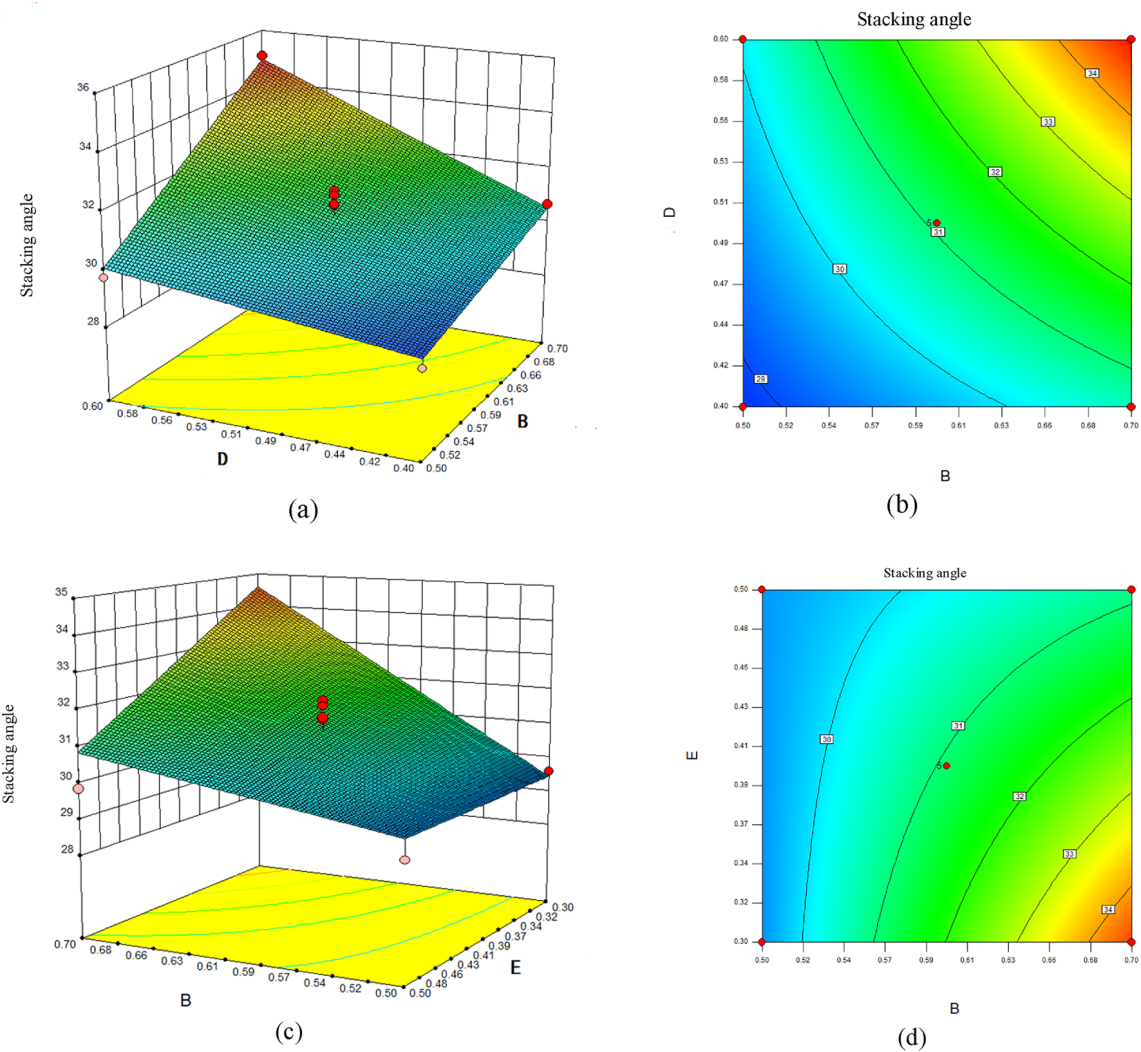
The Influence of Interaction Factors.

Based on the ANOVA results of the regression model, interaction terms involving B and D and B and E had a noteworthy repercussion on stacking angle. The response surface slope's steepness further confirms the significance of these factor interactions. Moreover, the contour lines' shape indicated the strength of this interaction^39^. Response surface outcomes for the stacking angle of sandy soil are illustrated in Fig. 7.

When set E at 0.3, the response surface plot for the interaction between B and D is presented in Fig. 7a,b. It could be observed that the response surface curve for B was steeper contrasted to the increasing direction of D. The Contour density of B was higher than the density along the increasing direction of D, revealing that B had a crucial repercussion on the stacking angle. The contour shape appeared to be ellipsoidal, suggesting a notable interaction betwixt B and D. When D was held at a certain value, the stacking angle enhanced as B enhanced. Similarly, when set B at a specific value, the angle tended to enhance as D enhanced. Notably, when B took on an immense value, the angle showed a significant enhancement as D enhanced.

When set D at 0.5, the response surface plot for interaction between B and E is presented in Fig. 7c,d. The response surface curve for B was steeper than E's increasing direction. The Contour density of B was higher than the density along the increasing direction of E, revealing that B had a crucial repercussion on the stacking angle. The contour shape appeared to be ellipsoidal, suggesting a notable interaction betwixt B and E. When E was held at a specific value, the stacking angle enhanced as B enhanced. Similarly, when set B at a specific value, the angle tended to lessen as E enhanced. Notably, when B took on a significant value, the angle significantly lessened as E enhanced.

#### Comparison of machine learning models

**Figure 8:**
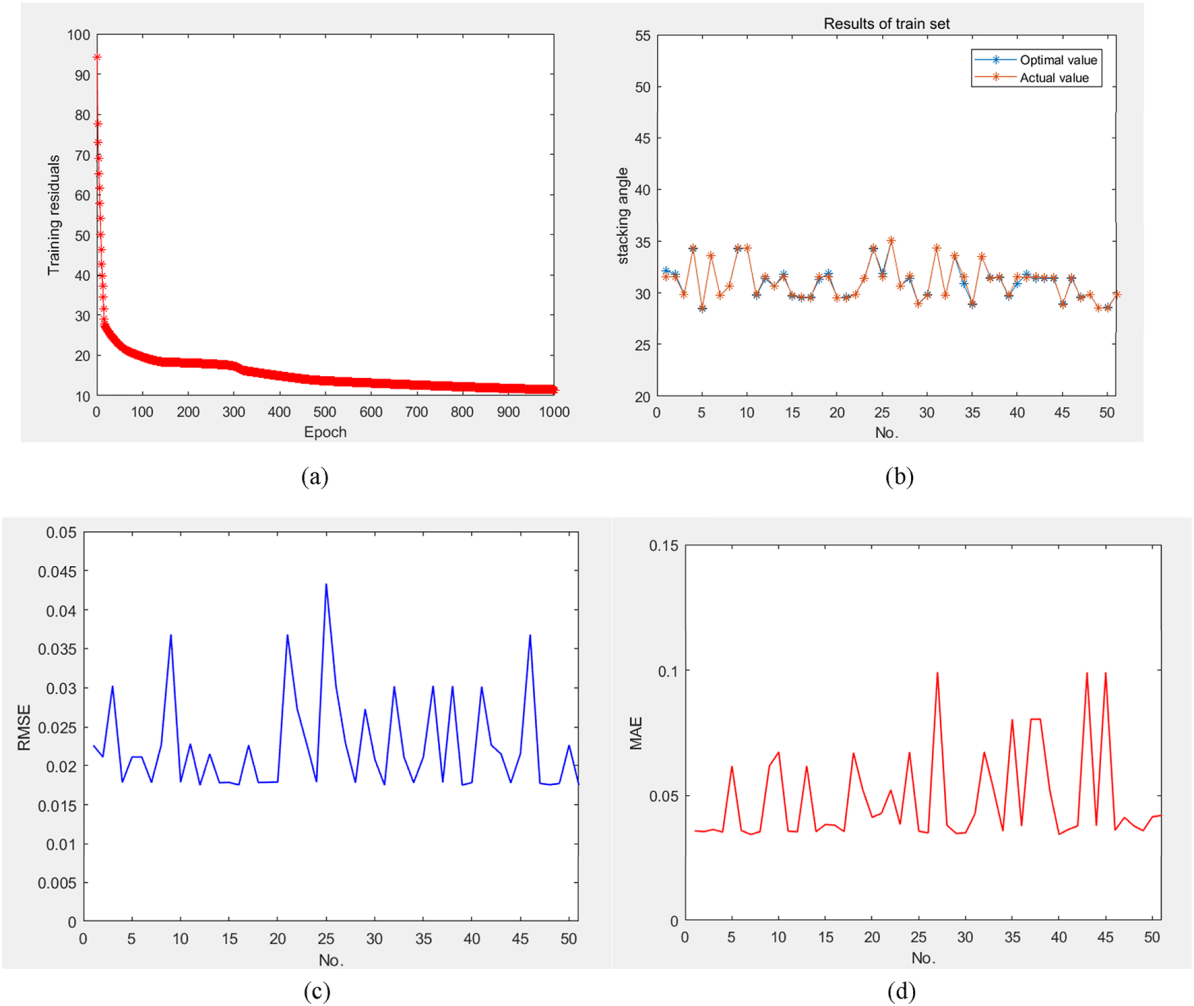
The results of train set.

This paper suggests conducting a comparative analysis of different machine learning algorithms to determine the most efficient fitting algorithm. Table 10 presents a comparative analysis of the non-linear algorithms. Figure 8a presents a visualization of WNN training residuals. The results demonstrate that the WNN surpasses the others in achieving lower relative errors. Besides, the BP algorithm registered a mean absolute error (MAE) of 0.28 and a root mean square error (RMSE) 0.64.

Meanwhile, the BP-GA algorithm showed improved performance with an MAE of 0.11 and an RMSE of 0.18. WNN algorithm outshone both, with the lowest recorded MAE of 0.068 and an RMSE of 0.035. Figure 8b illustrates the results of the train set, while Fig. 8c,d depict the RMSE of the training process and MAE of the training process, respectively. This outcome underscores the substantial limitations inherent in both BP and BP-GA algorithms about their precision in identifying optimal weights and thresholds. This excessive workload adversely impacts the accuracy and stability of its predictive outcomes.

This experiment has identified WNN as the most excellent optimization machine learning algorithm. Through analysis, it becomes evident that the training residual gradually decreases as the epoch progresses. However, the RMSE and MAE exhibit significant fluctuations throughout the training process.

### Comparison of non-linear tools

#### RSM parameter optimization

Design Expert was harnessed to determine the finest values of three significant factors by solving an optimized regression equation. The optimal value of B was 0.63, D was 0.55, and E was 0.43. Median values of non-significant parameters were chosen (A was 0.4, C was 0.2, and F was 0.22). The abovementioned parameters were adopted to validate the accuracy of optimal parameter amalgamation, and other parameter configurations remained unchanged. EDEM was applied to execute simulation tests on stacking angle. The average value acquired from three repeated simulation tests was 31.75°. Its relative error from the actual value of 32.15° was 1.24%. Optimization constraints for solving it are defined as,

17 $$\left\{ \begin{gathered} \alpha \to 32.15^\circ \hfill \\ s.t.\left\{ \begin{gathered} 0.2 \le B \le 0.7, \hfill \\ 0.2 \le D \le 0.6, \hfill \\ 0.3 \le E \le 0.8 \hfill \\ \end{gathered} \right. \hfill \\ \end{gathered} \right.$$

#### WNN parameter optimization

During the training process, the parameters of WNN are set as follows. The first learning rate is 0.001, the second is 0.01. The number of hidden layers is 7, and the training epochs are 100. Through WNN optimization, the stacking angle was determined to be 32.1°, exhibiting a relative error of 0.15% compared to the actual stacking angle of 32.15°. By analyzing the coordinates and conducting calculations, it was ascertained that B equaled 0.71, D equaled 0.62, and E equaled 0.41 during this instance. Through optimization utilizing BP-GA, the stacking angle achieved was 32.04°. By solely applying BP optimization, the stacking angle was measured at 31.45°. WNN demonstrates higher prediction accuracy than other non-linear tools and exhibits a more minor optimized stacking angle prediction error than other non-linear tools.

## Conclusions

It is concluded that the calibration of contact parameters for sandy soil in Xinjiang provides a theoretical foundation for parameter optimization of economic crop mechanized production in Xinjiang desert areas. From the above content, details of the conclusion can be reached as follows.

This study identified the key influencing factors based on their respective *P*-values. Factors with a *P*-value of less than 0.05 were deemed to have a significant impact. According to the Plackett–Burman design for screening tests, it has been determined that the contact parameters significantly influence the stacking angle. These parameters include sandy soil-steel static friction coefficient, sandy soil-steel restitution coefficient and sandy soil-sandy soil static friction coefficient. According to ANOVA results, the interaction terms of sandy soil-sandy soil static friction coefficient and sandy soil-steel static friction coefficient and the interaction term of sandy soil-sandy soil static friction coefficient and sandy soil-steel restitution coefficient had crucial impacts. Utilize the three key factors as input and subsequently employ nonlinear tools to optimize the stacking angle data. In WNNs, the wavelet transform facilitates multi-resolution analysis, enabling the simultaneous processing of both local and global characteristics of stacking angle data. The wavelet functions possess superior time–frequency localization properties, effectively capturing the data's transient variations and local nuances. This characteristic is advantageous for analyzing nonlinear characteristics inherent in stacking angle datasets. Due to the above characteristics, WNN outperforms other nonlinear tools in the experiment. In RSM, the relative error with physical test value is 1.24%. In BP-GA, the relative error with physical test value is 0.34%. In BP, the relative error with physical test value is 2.18%. In WNN, the relative error with physical test value is 0.15%.

Nevertheless, attaining a more accurate value remains a significant challenge during the optimization process of WNN models. This challenge has also been noted in previous studies^40^. To mitigate computational costs, in the future, this study will enhance the evaluation metrics for machine learning algorithms and optimize WNN and integrating it with other algorithms. Additionally, the unique distribution and complexity of the dataset imply that the model and hyperparameter settings are tailored to the sample data utilized in this study. To ensure broader applicability for different kinds of datasets, additional experiments are imperative to expand the scope of the dataset in future research.

## Data Availability

The datasets generated and/or analysed during the current study are not publicly available due permission issues related to soil collection sites but are available from the corresponding author on reasonable request. Example from: https://www.nature.com/articles/s41559-017-0329-x.
